# 2-{[4-(Diethyl­amino)­phen­yl]imino­methyl}-4,6-diiodo­phenol

**DOI:** 10.1107/S160053681004417X

**Published:** 2010-11-06

**Authors:** A. Thirugnan Sundar, G. Rajagopal, S. Murugavel, A. Subbiah Pandi

**Affiliations:** a Department of Chemistry, Dr Rangarajan Dr Sakunthala Engineering College, Chennai 600 062, India; b Department of Chemistry, Government Arts College, Melur, Madurai 625 106, India; c Department of Physics, Thanthai Periyar Government Institute of Technology, Vellore 632 002, India; d Department of Physics, Presidency College (Autonomous), Chennai 600 005, India

## Abstract

In the title compound, C_17_H_18_I_2_N_2_O, the dihedral angle between the aromatic rings is 5.4 (1)°. An intra­molecular O—H⋯N hydrogen bond generates an *S*(6) ring motif. The crystal packing is stabilized by C—H⋯π and π–π inter­actions [centroid–centroid distance = 3.697 (1) Å].

## Related literature

For Schiff base compounds in coordination chemistry, see: Weber *et al.* (2007 ▶); Chen *et al.* (2008 ▶). For their role in biological processes, see: May *et al.* (2004 ▶). For hydrogen-bond motifs, see: Bernstein *et al.* (1995 ▶). For related structures, see: Manvizhi *et al.* (2010 ▶).
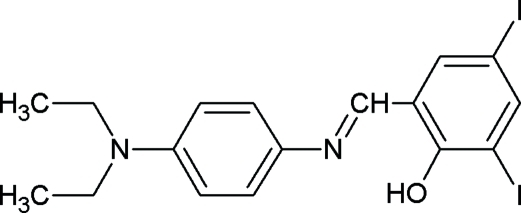


## Experimental

### 

#### Crystal data

C_17_H_18_I_2_N_2_O
*M* *_r_* = 520.13
Monoclinic, 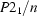

*a* = 11.5562 (5) Å
*b* = 11.1325 (5) Å
*c* = 15.1207 (6) Å
β = 111.958 (2)°
*V* = 1804.15 (13) Å^3^
*Z* = 4
Mo *K*α radiation
μ = 3.49 mm^−1^
*T* = 293 K
0.24 × 0.22 × 0.16 mm

#### Data collection

Bruker APEXII CCD diffractometer
Absorption correction: multi-scan (*SADABS*; Sheldrick, 1996 ▶) *T*_min_ = 0.450, *T*_max_ = 0.572
26163 measured reflections
7041 independent reflections
4445 reflections with *I* > 2σ(*I*)
*R*_int_ = 0.026

#### Refinement

*R*[*F* ^2^ > 2σ(*F* ^2^)] = 0.036
*wR*(*F* ^2^) = 0.105
*S* = 1.01
7041 reflections
202 parameters
H-atom parameters constrained
Δρ_max_ = 1.14 e Å^−3^
Δρ_min_ = −1.24 e Å^−3^

### 

Data collection: *APEX2* (Bruker, 2004 ▶); cell refinement: *SAINT* (Bruker, 2004 ▶); data reduction: *XPREP* (Bruker, 2004 ▶); program(s) used to solve structure: *SHELXS97* (Sheldrick, 2008 ▶); program(s) used to refine structure: *SHELXL97* (Sheldrick, 2008 ▶); molecular graphics: *ORTEP-3* (Farrugia, 1997 ▶); software used to prepare material for publication: *SHELXL97* and *PLATON* (Spek, 2009 ▶).

## Supplementary Material

Crystal structure: contains datablocks global, I. DOI: 10.1107/S160053681004417X/bt5392sup1.cif

Structure factors: contains datablocks I. DOI: 10.1107/S160053681004417X/bt5392Isup2.hkl

Additional supplementary materials:  crystallographic information; 3D view; checkCIF report

## Figures and Tables

**Table 1:** Hydrogen-bond geometry (Å, °) *Cg*1 is the centroid of the C8–C13 ring.

*D*—H⋯*A*	*D*—H	H⋯*A*	*D*⋯*A*	*D*—H⋯*A*
O1—H1⋯N1	0.82	1.86	2.592 (3)	148
C16—H16*B*⋯*Cg*1^i^^i^	0.97	2.94	3.845 (4)	155

Symmetry code: (i) 
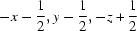
.

**Fig. 1.**
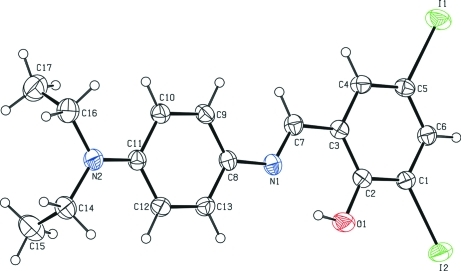
The molecular structure of the title compound with the atom numbering scheme. Displacement ellipsoids are drawn at the 30% probability level. H atoms are presented as a small cycles of arbitrary radius.

**Fig. 2.**
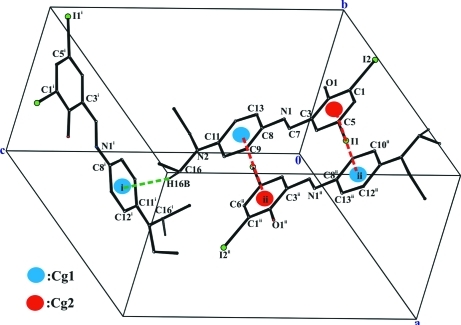
C-H···π and π-π interactions (dotted lines) in the title compound. Cg1 and Cg2 denote the centroids of the C8-C13 ring and C1-C6 ring, respectively. [Symmetry code: (i) -1/2-x, -1/2+y, 1/2-z; (ii) -x, -y, -z.]

## supplementary crystallographic information

### Comment

Schiff base compounds have received considerable attention for many years, primarily due to their importance in the development of coordination chemistry related to magnetism (Weber *et al.*, 2007), catalysis (Chen *et al.*, 2008) and biological processes (May *et al.*, 2004). Against this background, and in order to obtain detailed information on molecular conformations in the solid state, an X-ray study of the title compound has been carried out.

The molecular structure is illustrated in Fig. 1. The geometric parameters of the title molecule agrees well with those reported for a similar structure (Manvizhi *et al.*, 2010).  The dihedral angle between the aromatic rings is 5.4 (1)°, showing that both the rings  are almost coplanar.

In addition to the van der Waals interactions, the crystal packing is stabilized by  C-H···π hydrogen bonds as well as by π-π interactions. The intramolecular O-H···N hydrogen bond generates an S(6) ring motif (Bernstein *et al.*, 1995). The crystal packing (Fig. 2) is stabilized by C-H···π interactions between a H16B atom and a neighbouring ring, with a C16-H16B···Cg1^i^ separation of 2.94 Å (Fig. 2 and Table 1; Cg1 is the centroid of the C8-C13 ring ring, symmetry code as in Fig. 2). The molecular packing (Fig. 2) is further stabilized by π-π interactions with a Cg1···Cg2^ii^ and a Cg2···Cg1^ii^ separation of 3.697 (1)Å and 3.697 (1)Å, respectively (Fig. 2; Cg1 and Cg2 are the centroids of the C8-C13 benzene ring and C1-C6 benzene ring, respectively, symmetry code as in Table 1).

### Experimental

An ethanoic solution (30 ml) and N,N-diethyl aniline (10 mmol) was magnetically stirred in a round bottom flask followed by dropwise addition of 3,5-diiodosalicylaldehyde (10 mmol). The reaction mixture was then refluxed for two hours and upon cooling to 273K a yellow crystalline solid precipitated from the mixture. Single yellow crystals were obtained, filtered off, washed with ice cold ethanol and air dried.

### Refinement

All the H atoms were positioned geometrically, with O-H = 0.82 Å and and C-H = 0.93 - 0.98 Å and constrained to ride on their parent atom, with *U*_iso_(H)=1.2*U*_eq_.

### Crystal data

**Table:**

C_17_H_18_I_2_N_2_O	*F*(000) = 992
*M**_r_* = 520.13	*D*_x_ = 1.915 Mg m^−^^3^
Monoclinic, *P*2_1_/*n*	Mo *K*α radiation, λ = 0.71073 Å
Hall symbol: -P 2yn	Cell parameters from 7076 reflections
*a* = 11.5562 (5) Å	θ = 1.9–33.5°
*b* = 11.1325 (5) Å	µ = 3.49 mm^−^^1^
*c* = 15.1207 (6) Å	*T* = 293 K
β = 111.958 (2)°	Block, yellow
*V* = 1804.15 (13) Å^3^	0.24 × 0.22 × 0.16 mm
*Z* = 4	

### Data collection

**Table:**

Bruker APEXII CCD diffractometer	7041 independent reflections
Radiation source: fine-focus sealed tube	4445 reflections with *I* > 2σ(*I*)
graphite	*R*_int_ = 0.026
Detector resolution: 10.0 pixels mm^-1^	θ_max_ = 33.5°, θ_min_ = 1.9°
ω scans	*h* = −16→17
Absorption correction: multi-scan (*SADABS*; Sheldrick, 1996)	*k* = −10→17
*T*_min_ = 0.450, *T*_max_ = 0.572	*l* = −23→20
26163 measured reflections	

### Refinement

**Table:**

Refinement on *F*^2^	Secondary atom site location: difference Fourier map
Least-squares matrix: full	Hydrogen site location: inferred from neighbouring sites
*R*[*F*^2^ > 2σ(*F*^2^)] = 0.036	H-atom parameters constrained
*wR*(*F*^2^) = 0.105	*w* = 1/[σ^2^(*F*_o_^2^) + (0.0437*P*)^2^ + 1.1802*P*] where *P* = (*F*_o_^2^ + 2*F*_c_^2^)/3
*S* = 1.01	(Δ/σ)_max_ < 0.001
7041 reflections	Δρ_max_ = 1.14 e Å^−^^3^
202 parameters	Δρ_min_ = −1.24 e Å^−^^3^
0 restraints	Extinction correction: *SHELXL97* (Sheldrick, 2008), Fc^*^=kFc[1+0.001xFc^2^λ^3^/sin(2θ)]^-1/4^
Primary atom site location: structure-invariant direct methods	Extinction coefficient: 0.00078 (19)

### Special details

**Table:**

Geometry. All esds (except the esd in the dihedral angle between two l.s. planes) are estimated using the full covariance matrix. The cell esds are taken into account individually in the estimation of esds in distances, angles and torsion angles; correlations between esds in cell parameters are only used when they are defined by crystal symmetry. An approximate (isotropic) treatment of cell esds is used for estimating esds involving l.s. planes.
Refinement. Refinement of F^2^ against ALL reflections. The weighted R-factor wR and goodness of fit S are based on F^2^, conventional R-factors R are based on F, with F set to zero for negative F^2^. The threshold expression of F^2^ > 2sigma(F^2^) is used only for calculating R-factors(gt) etc. and is not relevant to the choice of reflections for refinement. R-factors based on F^2^ are statistically about twice as large as those based on F, and R- factors based on ALL data will be even larger.

### Fractional atomic coordinates and isotropic or equivalent isotropic displacement parameters (Å^2^)

**Table:**

	*x*	*y*	*z*	*U*_iso_*/*U*_eq_	
I1	0.33796 (2)	0.47955 (2)	0.048244 (18)	0.06508 (9)	
I2	−0.17213 (2)	0.45975 (3)	−0.257569 (17)	0.07853 (11)	
O1	−0.18770 (18)	0.2470 (2)	−0.12546 (15)	0.0561 (5)	
H1	−0.1935	0.1911	−0.0920	0.084*	
N1	−0.1205 (2)	0.10225 (19)	0.01926 (16)	0.0424 (5)	
N2	−0.2604 (2)	−0.2797 (2)	0.20106 (18)	0.0502 (5)	
C1	−0.0432 (2)	0.3956 (2)	−0.12942 (18)	0.0418 (5)	
C2	−0.0754 (2)	0.2987 (2)	−0.08510 (19)	0.0401 (5)	
C3	0.0127 (2)	0.2571 (2)	0.00140 (18)	0.0384 (5)	
C4	0.1298 (2)	0.3106 (2)	0.0396 (2)	0.0434 (5)	
H4	0.1882	0.2827	0.0969	0.052*	
C5	0.1596 (2)	0.4050 (2)	−0.00723 (19)	0.0413 (5)	
C6	0.0732 (2)	0.4483 (2)	−0.09115 (19)	0.0424 (5)	
H6	0.0931	0.5128	−0.1220	0.051*	
C7	−0.0155 (3)	0.1572 (2)	0.0516 (2)	0.0435 (5)	
H7	0.0447	0.1321	0.1091	0.052*	
C8	−0.1490 (2)	0.0059 (2)	0.06823 (19)	0.0393 (5)	
C9	−0.0659 (2)	−0.0478 (2)	0.1498 (2)	0.0448 (6)	
H9	0.0160	−0.0206	0.1753	0.054*	
C10	−0.1022 (3)	−0.1412 (3)	0.1942 (2)	0.0460 (6)	
H10	−0.0442	−0.1752	0.2490	0.055*	
C11	−0.2248 (2)	−0.1854 (2)	0.15812 (19)	0.0414 (5)	
C12	−0.3081 (3)	−0.1300 (3)	0.0757 (2)	0.0474 (6)	
H12	−0.3906	−0.1557	0.0501	0.057*	
C13	−0.2698 (3)	−0.0380 (2)	0.03204 (19)	0.0442 (6)	
H13	−0.3268	−0.0044	−0.0234	0.053*	
C14	−0.3909 (3)	−0.3145 (3)	0.1708 (2)	0.0564 (7)	
H14A	−0.3951	−0.3952	0.1936	0.068*	
H14B	−0.4266	−0.3168	0.1017	0.068*	
C15	−0.4696 (4)	−0.2328 (4)	0.2052 (3)	0.0820 (12)	
H15A	−0.4382	−0.2334	0.2737	0.123*	
H15B	−0.5544	−0.2605	0.1808	0.123*	
H15C	−0.4662	−0.1525	0.1832	0.123*	
C16	−0.1740 (3)	−0.3316 (3)	0.2895 (3)	0.0681 (9)	
H16A	−0.0935	−0.3421	0.2842	0.082*	
H16B	−0.2042	−0.4105	0.2977	0.082*	
C17	−0.1568 (4)	−0.2583 (5)	0.3760 (3)	0.0930 (15)	
H17A	−0.1348	−0.1777	0.3662	0.140*	
H17B	−0.0914	−0.2926	0.4300	0.140*	
H17C	−0.2331	−0.2575	0.3874	0.140*	

### Atomic displacement parameters (Å^2^)

**Table:**

	*U*^11^	*U*^22^	*U*^33^	*U*^12^	*U*^13^	*U*^23^
I1	0.04530 (12)	0.06753 (15)	0.07159 (16)	−0.01936 (9)	0.00944 (10)	−0.00041 (10)
I2	0.05227 (14)	0.1088 (2)	0.05720 (15)	−0.01130 (12)	0.00056 (10)	0.03482 (13)
O1	0.0423 (10)	0.0597 (12)	0.0577 (12)	−0.0140 (9)	0.0090 (9)	0.0060 (10)
N1	0.0457 (12)	0.0380 (10)	0.0479 (12)	−0.0021 (9)	0.0227 (10)	0.0008 (9)
N2	0.0510 (13)	0.0495 (13)	0.0506 (14)	−0.0104 (10)	0.0194 (11)	0.0062 (11)
C1	0.0391 (12)	0.0445 (13)	0.0393 (13)	−0.0014 (10)	0.0117 (10)	0.0037 (10)
C2	0.0373 (12)	0.0402 (12)	0.0435 (13)	−0.0012 (9)	0.0157 (10)	−0.0027 (10)
C3	0.0385 (12)	0.0336 (11)	0.0428 (13)	−0.0014 (9)	0.0149 (10)	−0.0011 (9)
C4	0.0400 (13)	0.0404 (12)	0.0442 (14)	−0.0019 (10)	0.0092 (10)	0.0023 (11)
C5	0.0365 (12)	0.0382 (12)	0.0462 (14)	−0.0050 (9)	0.0121 (10)	−0.0025 (10)
C6	0.0445 (14)	0.0386 (12)	0.0440 (14)	−0.0029 (10)	0.0166 (11)	0.0029 (10)
C7	0.0480 (14)	0.0373 (12)	0.0475 (14)	−0.0004 (10)	0.0206 (12)	0.0035 (11)
C8	0.0419 (13)	0.0358 (11)	0.0437 (13)	−0.0019 (9)	0.0201 (11)	−0.0004 (10)
C9	0.0365 (12)	0.0467 (14)	0.0528 (16)	−0.0041 (10)	0.0186 (11)	0.0022 (12)
C10	0.0421 (13)	0.0478 (14)	0.0483 (15)	−0.0003 (11)	0.0172 (11)	0.0065 (12)
C11	0.0449 (13)	0.0394 (12)	0.0423 (13)	−0.0050 (10)	0.0191 (11)	−0.0015 (10)
C12	0.0428 (13)	0.0521 (15)	0.0434 (14)	−0.0138 (11)	0.0115 (11)	−0.0024 (12)
C13	0.0437 (13)	0.0476 (14)	0.0380 (13)	−0.0064 (11)	0.0115 (11)	−0.0003 (11)
C14	0.0550 (17)	0.0511 (16)	0.0634 (19)	−0.0177 (13)	0.0225 (15)	0.0013 (14)
C15	0.059 (2)	0.096 (3)	0.098 (3)	−0.009 (2)	0.037 (2)	−0.010 (2)
C16	0.066 (2)	0.067 (2)	0.071 (2)	−0.0094 (17)	0.0248 (17)	0.0222 (18)
C17	0.089 (3)	0.123 (4)	0.061 (2)	−0.037 (3)	0.020 (2)	0.004 (2)

### Geometric parameters (Å, °)

**Table:**

I1—C5	2.085 (2)	C9—C10	1.385 (4)
I2—C1	2.080 (3)	C9—H9	0.9300
O1—C2	1.340 (3)	C10—C11	1.403 (4)
O1—H1	0.8200	C10—H10	0.9300
N1—C7	1.281 (3)	C11—C12	1.401 (4)
N1—C8	1.411 (3)	C12—C13	1.378 (4)
N2—C11	1.375 (3)	C12—H12	0.9300
N2—C14	1.456 (4)	C13—H13	0.9300
N2—C16	1.457 (4)	C14—C15	1.510 (5)
C1—C6	1.381 (4)	C14—H14A	0.9700
C1—C2	1.391 (4)	C14—H14B	0.9700
C2—C3	1.402 (4)	C15—H15A	0.9600
C3—C4	1.391 (3)	C15—H15B	0.9600
C3—C7	1.450 (3)	C15—H15C	0.9600
C4—C5	1.381 (4)	C16—C17	1.489 (6)
C4—H4	0.9300	C16—H16A	0.9700
C5—C6	1.376 (4)	C16—H16B	0.9700
C6—H6	0.9300	C17—H17A	0.9600
C7—H7	0.9300	C17—H17B	0.9600
C8—C9	1.383 (4)	C17—H17C	0.9600
C8—C13	1.384 (4)		
			
C2—O1—H1	109.5	C11—C10—H10	119.4
C7—N1—C8	122.5 (2)	N2—C11—C12	121.9 (2)
C11—N2—C14	120.8 (2)	N2—C11—C10	121.4 (3)
C11—N2—C16	120.9 (2)	C12—C11—C10	116.7 (2)
C14—N2—C16	117.2 (2)	C13—C12—C11	121.1 (2)
C6—C1—C2	121.4 (2)	C13—C12—H12	119.5
C6—C1—I2	119.38 (19)	C11—C12—H12	119.5
C2—C1—I2	119.18 (19)	C12—C13—C8	122.0 (3)
O1—C2—C1	120.1 (2)	C12—C13—H13	119.0
O1—C2—C3	121.6 (2)	C8—C13—H13	119.0
C1—C2—C3	118.3 (2)	N2—C14—C15	114.7 (3)
C4—C3—C2	120.0 (2)	N2—C14—H14A	108.6
C4—C3—C7	119.0 (2)	C15—C14—H14A	108.6
C2—C3—C7	121.0 (2)	N2—C14—H14B	108.6
C5—C4—C3	120.2 (2)	C15—C14—H14B	108.6
C5—C4—H4	119.9	H14A—C14—H14B	107.6
C3—C4—H4	119.9	C14—C15—H15A	109.5
C6—C5—C4	120.4 (2)	C14—C15—H15B	109.5
C6—C5—I1	119.91 (19)	H15A—C15—H15B	109.5
C4—C5—I1	119.71 (19)	C14—C15—H15C	109.5
C5—C6—C1	119.6 (2)	H15A—C15—H15C	109.5
C5—C6—H6	120.2	H15B—C15—H15C	109.5
C1—C6—H6	120.2	N2—C16—C17	114.2 (3)
N1—C7—C3	122.2 (3)	N2—C16—H16A	108.7
N1—C7—H7	118.9	C17—C16—H16A	108.7
C3—C7—H7	118.9	N2—C16—H16B	108.7
C9—C8—C13	117.5 (2)	C17—C16—H16B	108.7
C9—C8—N1	125.2 (2)	H16A—C16—H16B	107.6
C13—C8—N1	117.4 (2)	C16—C17—H17A	109.5
C8—C9—C10	121.4 (3)	C16—C17—H17B	109.5
C8—C9—H9	119.3	H17A—C17—H17B	109.5
C10—C9—H9	119.3	C16—C17—H17C	109.5
C9—C10—C11	121.3 (3)	H17A—C17—H17C	109.5
C9—C10—H10	119.4	H17B—C17—H17C	109.5
			
C6—C1—C2—O1	−177.9 (2)	C7—N1—C8—C13	173.1 (2)
I2—C1—C2—O1	0.8 (3)	C13—C8—C9—C10	−0.6 (4)
C6—C1—C2—C3	1.6 (4)	N1—C8—C9—C10	179.1 (3)
I2—C1—C2—C3	−179.80 (18)	C8—C9—C10—C11	0.2 (4)
O1—C2—C3—C4	177.9 (2)	C14—N2—C11—C12	−9.1 (4)
C1—C2—C3—C4	−1.5 (4)	C16—N2—C11—C12	−176.7 (3)
O1—C2—C3—C7	−1.1 (4)	C14—N2—C11—C10	171.8 (3)
C1—C2—C3—C7	179.5 (2)	C16—N2—C11—C10	4.3 (4)
C2—C3—C4—C5	0.2 (4)	C9—C10—C11—N2	178.7 (3)
C7—C3—C4—C5	179.2 (2)	C9—C10—C11—C12	−0.4 (4)
C3—C4—C5—C6	1.1 (4)	N2—C11—C12—C13	−178.0 (3)
C3—C4—C5—I1	−177.55 (19)	C10—C11—C12—C13	1.1 (4)
C4—C5—C6—C1	−1.1 (4)	C11—C12—C13—C8	−1.6 (4)
I1—C5—C6—C1	177.6 (2)	C9—C8—C13—C12	1.3 (4)
C2—C1—C6—C5	−0.3 (4)	N1—C8—C13—C12	−178.4 (2)
I2—C1—C6—C5	−178.9 (2)	C11—N2—C14—C15	−76.2 (4)
C8—N1—C7—C3	−179.3 (2)	C16—N2—C14—C15	91.8 (4)
C4—C3—C7—N1	−178.5 (2)	C11—N2—C16—C17	76.8 (4)
C2—C3—C7—N1	0.6 (4)	C14—N2—C16—C17	−91.2 (4)
C7—N1—C8—C9	−6.6 (4)		

### Hydrogen-bond geometry (Å, °)

**Table:**

Cg1 is the centroid of the C8–C13 ring.

**Table:**

*D*—H···*A*	*D*—H	H···*A*	*D*···*A*	*D*—H···*A*
O1—H1···N1	0.82	1.86	2.592 (3)	148
C16—H16B···Cg1^i^^i^	0.97	2.94	3.845 (4)	155

Symmetry codes:  (i) −*x*−1/2, *y*−1/2, −*z*+1/2.
